# Comparing clinical effects of marbofloxacin and gamithromycin in goat kids with pneumonia

**DOI:** 10.4102/jsava.v89i0.1558

**Published:** 2018-06-20

**Authors:** Yigit Kacar, Hasan Batmaz, Ozge E. Yilmaz, Zafer Mecitoglu

**Affiliations:** 1 Department of Internal Medicine, Veterinary Faculty, Uludag University, Turkey; 2 Department of Microbiology, Veterinary Faculty, Uludag University, Turkey

## Abstract

The aim of this study was to compare the clinical efficacy of a single-dose of gamithromycin (GM) or marbofloxacin (MR) in kids with naturally occurring pneumonia. Thirty-six kids, aged 2–2.5 months, with body weight ranging from 12 kg to 18 kg were presented with clinical signs of pneumonia. The most prominent clinical findings were an increase in the respiratory rate, crackling lung sounds on auscultation, coughing, nasal discharge and an increased rectal temperature. *Mannheimia haemolytica* and *Mycoplasma* spp. colonies were isolated from microbiological examination of six transtracheal washes and lung tissues of one necropsied kid. The severity of pneumonia was evaluated by using cumulative clinical score (CCS). The CCS of the 36 kids used in the study were four and above. Kids were randomly divided into two equal groups; the GM group received a single subcutaneous dose of GM at a dosage of 6 mg/kg and the MR group received MR intramuscularly at a dosage of 8 mg/kg as a single-dose. No side effects related to the drugs were detected in either group. All 36 kids were clinically examined 3 weeks after the initiation of the treatment. Clinical signs in both groups were almost completely absent at the end of the study. A single administration of GM or MR was successfully used in the treatment of kids with pneumonia.

## Introduction

Pneumonia caused by mainly *Mannheimia haemolytica, Pasteurella multocida, Bibersteinia trehalosi* and *Mycoplasma* spp. (*Mycoplasma ovipneumoniae* and *Mycoplasma arginini*) (Brogden, Lehmkuhl & Cutlip 1998; Clothier, Kinyon & Griffith 2012; Fleming 2009; Matthews 2009; Oros et al. 1997; Ozbey & Muz 2004) is a common health problem in goats, especially in goat kids. Poor management and suboptimal factors such as inadequate colostrum intake, inadequate nutrition, inadequate ventilation, high humidity, and keeping different age groups together are important environmental factors that increase the susceptibility of animals to pneumonia (Fleming 2009; Matthews 2009). In goat kids, pneumonic pasteurellosis caused by *M. haemolytica* and *P. multocida* usually affects animals younger than 3–4 months of age with higher mortality rates in kids aged 2–3 weeks (Batmaz 2013; Fleming 2009).

The most widely used antibiotics for treatment of pneumonia in cattle are macrolides and quinolones. Beta lactams and florfenicol are also commonly used antibiotics for the treatment of pneumonia in ruminants (Apley & Coetzee 2013). Procaine penicillin, amoxicillin, ampicillin, oxytetracycline, erythromycin, tylosin, enrofloxacin and new molecules such as ceftiofur, tulathromycin and florfenicol are antibiotics widely used in the treatment of pneumonia in goats (Clothier et al. 2012; Elitok et al. 2015; Fleming 2009; Matthews 2009). Quinolones are commonly used antibiotics in pneumonia owing to their broad spectrum and higher penetration rates into lung tissue (Apley & Coetzee 2013; DeDonder et al. 2016). A quinolone antibiotic marbofloxacin (MR) has been demonstrated to be effective in the treatment of contagious caprine pleuropneumonia (Balikçi et al. 2008). Marbofloxacin was also successfully used in lambs with pneumonia caused by mixed infections of *M. haemolytica* and *Mycoplasma* spp. at a dosage of 2–3 mg/kg for three consecutive days or 2 doses 3 days apart, respectively (Skoufos et al. 2007).

A single-dose of MR at a higher dosage rate is reported to be effective in the treatment of bovine respiratory disease (Grandemange et al. 2012). The pharmacokinetic evaluation of MR in goats demonstrated good tissue distribution and clearance when administered either intravenously or intramuscularly (Waxman et al. 2001). However, effectiveness of MR in pneumonia of goat kids has not been reported before.

Macrolide antibiotics are preferred for the treatment of pneumonia as they can reach high concentrations in lung tissue (DeDonder et al. 2016). Gamithromycin (GM) is a macrolide antibiotic licensed for treatment and control of bovine respiratory disease and is reported to be effective in the treatment of pneumonia caused by *M. hemolytica, P. multocida* and *Mycoplasma bovis* (Apley & Coetzee 2013; Baggott et al. 2011). Gamithromycin has been successfully used for whole flock antibiotic treatment against footrot of sheep caused by *Dichelobacter nodosus* (Forbes, Strobel & Stamphoj 2014). The therapeutic effects of GM in pneumonia in goat kids have not been reported.

The aim of this study was to evaluate the clinical efficacy of a single-dose of MR or GM in a naturally occurring outbreak of pneumonia in goat kids.

## Material and methods

The study was conducted in a goat flock that had approximately 100 Saanen-Turkish native mixed breed kids aged between 2.0 and 2.5 months. The owner of the flock reported that the kids had been suffering from a respiratory disease for the past 5 days. Despite treatment with oxytetracycline by the owner, five kids died. The flock was not vaccinated against respiratory diseases. A necropsy was conducted on a single kid that died.

All of the kids were clinically examined and respiratory rates, lung auscultation findings, laryngotracheal palpation results and rectal temperatures were recorded. Cumulative clinical scores (CCS) (Table 1) of all animals were obtained by modifying a clinical scoring system used for calves (Poulsen & McGuirk 2009) and lambs (Christodoulopoulos et al. 2002). Thirty-six kids with CCS scores of 4 and above that did not receive the oxytetracycline treatment were selected for the study. Kids were randomly assigned to two groups consisting of 18 animals. Animals in the GM group (*n* = 18) received a single subcutaneous dose of GM (Zactran^®^, Merial) at a dosage of 6 mg/kg or a single intramuscular dose of MR (Marbox^®^, Ceva) (MR group, *n* = 18) at a dosage of 8 mg/kg.

**TABLE 1:** Cumulative clinical scoring system used in the study.

Score	0	1	2
T (°C)	Normal	-	< 38.5 > 40.5
Respiratory rate (per min)	< 20	20–40	> 40
Lung auscultation	Normal	Mildly harsh sounds	Crackles and wheezes
Coughing	Absent	Induced	Spontaneous
Nasal discharge	Absent	Mild to moderate, serous to mucoid	Severe mucopurulent
Lacrimation	Absent	Mild to moderate	-
Conjunctival colour	Normal	Hyperemic	-
Cumulative clinical score	-	-	-

*Source*: Adapted from Christodoulopoulos, G., Warnick, L.D., Papaioannuo, N. & Fthenakis, G.C., 2002, ‘Tilmicosin administration to young lambs with respiratory infection: Safety and efficacy considerations’, *Journal of Veterinary Pharmacology and Therapeutics* 25, 393–397. https://doi.org/10.1046/j.1365-2885.2002.00433.x and Poulsen, K.P. & McGuirk, S.M., 2009, ‘Respiratory disease of the bovine neonate’, *Veterinary Clinics of North America: Food Animal Practice* 25, 121–137. https://doi.org/10.1016/j.cvfa.2008.10.007

Percutaneous transtracheal washing fluid samples of six sick kids were collected for microbiological examination as previously described (Ural et al. 2011). Lung samples of the necropsied kid were also collected for microbiological examination. Each transtracheal fluid sample was inoculated onto two 5% sheep blood agars (GBL, İstanbul, Turkey), two MacConkey agars (Merck, Darmstadt, Germany) and a *Mycoplasma* agar (Oxoid, Basingstoke, Hampshire, England). One blood agar and a MacConkey agar were incubated at 37 °C in an aerobic atmosphere, and the other blood agar, MacConkey agar, and *Mycoplasma* agar were incubated at 37 °C in a microaerophilic atmosphere. After 24–48 h, suspected colonies were examined according to gram stain and culture characteristics with a commercial biochemical test kit API 20 E (bioMérieux S.A., Marcy-l’Etoile, France). At the end of 5 days, *Mycoplasma* agar was removed from the microaerophilic incubator and then examined by stereomicroscope and *Mycoplasma* spp. colonies were observed. Three weeks after the beginning of treatment, 36 kids from both groups were re-evaluated and scored.

### Ethical consideration

This study was conducted on goat kids suffering from a natural outbreak of pneumonic pasteurellosis. All animals suffering from the disease were treated with antibiotics. None of the kids were left without treatment for consideration as a control group. None of the treated kids died.

## Results

The most prominent findings in the clinical examination of the 36 kids were the increased respiratory rate and the harsh sounds on auscultation of the lungs (Table 2). No side effects were observed after the single-dose administration of MR and GM. The mean CCS in both groups was 6.16 at the beginning of the study (Table 3). A CCS of 4 and 5 was evaluated as mild, 6 and 7 as moderate and 8–10 as severe pneumonia (Table 3).

**TABLE 2:** Detected clinical findings in 36 goat kids.

Clinical findings	*n*	%
**Increase in respiratory frequency**	**36**	**100.0**
Respiratory rate/min 20–40	23	63.8
Respiratory rate/min > 40	13	36.1
**Harsh sounds on lung auscultation**	**36**	**100.0**
Mildly harsh sounds	18	50.00
Markedly harsh sounds	18	50.00
**Cough**	**29**	**80.5**
Mild, generated	15	41.6
Spontaneous, prominent	14	38.8
**Nasal discharge**	**25**	**69.4**
Light-medium and sero-mucoid	10	27.7
Severe and mucopurulent	15	41.6
**Change in rectal temperature**	**15**	**41.6**
T > 40.5 °C	14	38.8
T < 38.5 °C	1	2.7
**Conjunctival hyperemia**	**14**	**38.8**
**Lacrimation**	**4**	**11.1**

Note: Data given in bold is the sum of the findings in the category.

T, temperature; C, celcius.

**TABLE 3:** Pre- and post-treatment cumulative clinical scores in both groups of kids with acute pneumonia.

Severity of pneumonia	CCS	Pre-treatment (Day 0)		Post-treatment (Day 21)	
		GM (*n* = 1)	MR (*n* = 1)	GM (*n* = 18)	MR (*n* = 18)
-	0	-	-	2	1.00
	1	-	-	4	4.00
	2	-	-	6	6.00
	3	-	-	4	4.00
Mild	4	3.00	5.00	-	-
	5	4.00	2.00	-	-
Moderate	6	4.00	1.00	-	-
	7	3.00	7.00	-	-
Severe	8	3.00	2.00	-	-
	9	0.00	0.00	-	-
	10	1.00	1.00	-	-
**Mean CCS**		**6.16**	**6.16**	**2**	**2.22**

CSS, cumulative clinical scores; GM, gamithromycin; MR, marbofloxacin.

**FIGURE 1:**
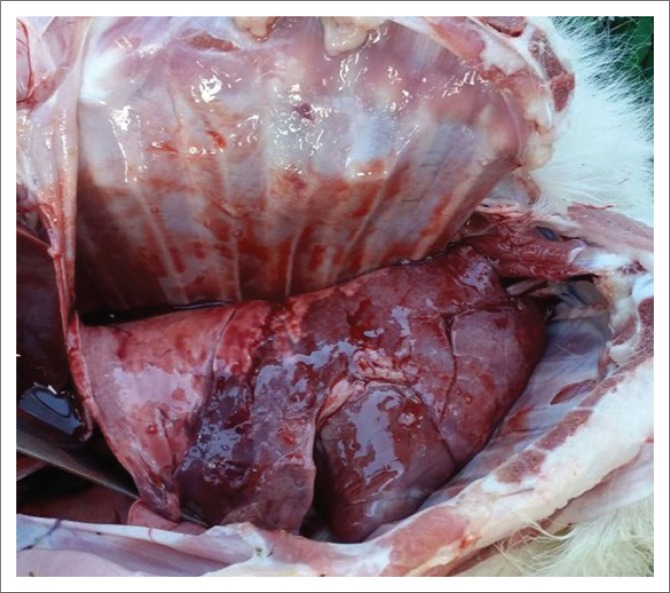
Dark red hepatisation or consolidation in the cranioventral lung lobes of the goat kid.

An autopsy conducted on the kid that died revealed dark red hepatisation/consolidation of the cranioventral lung lobes (Figure 1).

*M. haemolytica* and *Mycoplasma* spp. colonies were identified on microbiological examination of the lung and tracheal fluid samples of the necropsied animal. The same pathogens were also detected in the transtracheal fluid aspirates of six kids. A diagnosis of pneumonic pasteurellosis was based on the clinical, necropsy and microbiological findings.

Three weeks after the initiation of treatments, almost all of the goat kids of the GM and MR groups were completely healthy. However, despite the good overall condition and appetite, two kids from the GM group and three from the MR group were still suffering from mild pneumonia based on CCS scoring (Table 3). Mean CCS of the GM and MR groups were 2.00 and 2.22 at the end of the study, respectively. Differences between CCS before and 3 weeks after the treatment were 4.16 and 3.94 for GM and MR groups, respectively.

## Discussion

Pneumonia in goats are more prevalent in younger animals and the animals included in this study were 2.0- to 2.5-month-old kids. *M. haemolytica, P. multocida* and *Mycoplasms*a spp. are reported to be the major pathogens associated with pneumonia in goats (Brogden et al. 1998; Fleming 2009; Matthews 2009). In accordance with their findings, *M. hemolytica* and *Mycoplasma* spp. were also the pathogens isolated from goat kids suffering from pneumonia in this study. Prominent, harsh lung sounds; nasal discharge; and increased rectal temperature were the main clinical findings in a study conducted on goats with pneumonia caused by *P. multocida* (Sadeghian et al. 2011). Increased respiratory rates, harsh lung sounds as well as coughing and nasal discharges were the major clinical findings observed, while rectal temperatures of only 14 out of 36 kids were elevated before initiation of treatment.

Oxytetracycline and tylosin, separately or in combination, are antibiotics widely used for the treatment of pneumonia in goats (Fleming 2009). Because of secondary invaders such as *Trueperella pyogenes*, second-line treatment with antibiotics such as procaine penicillin, amoxicillin and ampicillin is advocated (Ribeiro et al. 2015). However, in recent years, extra label use of some antibiotics such as ceftiofur, enrofloxacin, tulathromycin and florfenicol has been studied for the treatment of pneumonia in goats (Clothier et al. 2012; Fleming 2009; Matthews 2009). Gamithromycin and MR have been used successfully in pneumonia treatments of cattle (Apley & Coetzee 2013); extralabel use of these drugs in goat kids gave acceptable results for the treatment of pneumonia caused by *Manheimia haemolytica* and *P. multocida* in this investigation. These medications also have the advantage of effectiveness in single-dose administration, which is labour efficient and more practical under extensive farming conditions.

Marbofloxacin is a quinolone antibiotic with bactericidal effects and GM is a macrolide antibiotic with bacteriostatic effects on bacteria. Both antibiotics are effective against *Mycoplasma* spp., gram-positive bacteria and gram-negative bacteria (Apley & Coetzee 2013; DeDonder et al. 2016). The recommended dosage regimen for MR for cattle is 2 mg/kg for 3–5 days. However, it is reported that MR, as a single-dose of 10 mg/kg, could be effective in order to limit the risk of development of resistance, as multiple dosing of 2 mg/kg could achieve mutant prevention concentration (MPC) only for a short time after every dose (Vallé et al. 2012). Based on this, we used MR as a single high dose as recommended for calves and pigs in previous studies (Grandemange et al. 2012, 2017).

The most important side effects of quinolones are damage to articular cartilage in young and rapidly growing animals owing to complexes forming between quinolones and magnesium (Polachek, Leibovitz & Dagan 2011). However, it is reported that quinolone-related cartilage damage is a species-specific phenomenon that is especially prevalent in primates, rats and canines (Sansone et al. 2009). Sansone et al. (2009) did not observe any cartilage damage in lambs treated with ciprofloxacin and gatifloxacin. Similarly, we did not observe any clinical side effects and/or lameness in either of the group**s** during the 3-week study period.

In this study, pneumonia in kids caused by mixed infections of *M. haemolytica* and *Mycoplasma* spp. was treated with one or the other of the study antibiotics. All kids survived in our study; however, two kids from the GM and three from the MR group were not totally cured and showed signs of mild pneumonia in the third week after the treatment. This may be because of delays in the initiation of the treatment, based on the normal rectal temperatures in 22 of 36 kids at the beginning of the study. Switching to other antibiotics could have been beneficial in these cases, as a range of antibiotics has been recommended for the treatment of calves with pneumonia caused by *M. hemolytica, P. multocida, Mycoplasma* spp. and *T. pyogenes* (Apley & Coetzee 2013).

Despite the demonstrated efficacy of a macrolide and a quinolone in this study, and despite registration for use in food-producing animals in several countries, these medically important antibiotics, in the current drive to control resistance to antibiotics, should not be used in food-producing animals. Both macrolides and quinolones are listed by the World Health Organization as critically important antibiotics in human medicine. In this study, these antibiotics were not used to prevent disease but to treat a natural outbreak of disease, where oxytetracycline was found to be ineffective, using a single-dose at a relatively high dosage rate.

## Conclusion

In conclusion, GM and MR were equally effective in the treatment of pneumonia in goat kids caused by a mixed infection of *M. hemolytica* and *Mycoplasma* spp. However, constant treatment is crucial to avoid chronicity.
